# Variable clinical characteristics and laboratory results in five patients with Chinese Good's syndrome (thymoma and hypogammaglobulinemia): an 8-year retrospective analysis in a university hospital in China

**DOI:** 10.1186/s12865-021-00441-9

**Published:** 2021-08-03

**Authors:** Jinyao Ni, Junwu Zhang, Yanxia Chen, Weizhong Wang, Jinlin Liu

**Affiliations:** 1 Department of Pathology, The First Affiliated Hospital of Wenzhou Medical University, Wenzhou, 325015 China; 2 Department of Clinical Laboratory , Wenzhou Hospital of Traditional Chinese Medicine Affiliated to Zhejiang Chinese Medical University, 9 Jiaowei Road, Wenzhou, 325000 China; 3 Department of Rheumatology, Zhejiang Provincial People’s Hospital, Hangzhou Medical College, 158 Shangtang Road, Hangzhou, 310014 China; 4 Department of Clinical Laboratory, Zhejiang Provincial People’s Hospital, Hangzhou Medical College, 158 Shangtang Road, Hangzhou, 310014 China

**Keywords:** Good's syndrome, Thymoma, Hypogammaglobulinemia

## Abstract

**Background:**

Good's syndrome (GS) is a rare secondary immunodeficiency disease presenting as thymoma and hypogammaglobulinemia. Due to its rarity, the diagnosis of GS is often missed.

**Methods:**

We used the hospital information system to retrospectively screen thymoma and hypogammaglobulinemia patients at the First Affiliated Hospital of Wenzhou Medical University from Apr 2012 to Apr 2020. The clinical, laboratory, treatment, and outcome data for these patients were collected and analyzed.

**Results:**

Among the 181 screened thymoma patients, 5 thymoma patients with hypogammaglobulinemia were identified; 3 patients had confirmed diagnoses of GS, and the other 2 did not have a diagnosis of GS recorded in the hospital information system. A retrospective review of the clinical characteristics, laboratory results, and follow-up data for these 2 undiagnosed patients confirmed the diagnosis of GS. All 5 GS patients presented with pneumonia, 2 patients presented with recurrent skin abscesses, 2 patients presented with recurrent cough and expectoration, 1 patient presented with recurrent oral lichen planus and diarrhea, and 1 patient presented with tuberculosis and granulomatous epididymitis. In the years after the diagnosis of hypogammaglobulinemia with mild symptoms, all 5 patients had received irregular intravenous immunoglobulin (IVIG) treatment. As the course of the disease progressed, the clinical symptoms of all patients worsened, but the symptoms were partly resolved with IVIG in these patients. However, 4 patients died due to comorbidities.

**Conclusion:**

GS should be investigated as a possible diagnosis in thymoma patients who present with hypogammaglobulinemia, especially those with recurrent opportunistic infections, recurrent skin abscesses, chronic diarrhea, or recurrent lichen planus.

## Background

Adult-onset hypogammaglobulinemia associated with thymoma was first described by Good et al. in 1954 and is known as Good’s syndrome (GS) [1]. GS is a rare disease that is difficult to diagnose preoperatively; it is characterized by thymoma, hypogammaglobulinemia, low or absent B cells, and decreased T cells [2]. The main clinical manifestations include thymoma, infection, gastrointestinal manifestations (diarrhea), and autoimmune manifestations (myasthenia gravis, pure red cell aplasia, and oral lichen planus) [1]. However, GS is difficult to diagnose due to its rarity, lack of typical symptoms, and lack of a comprehensive understanding of this disease [3].

Importantly, GS should be considered in every patient with a history of thymoma and recurrent infections. Immunologic evaluation of these patients, including measurements of serum immunoglobulin levels and B cells, should be performed. An appropriate diagnostic immunological work-up should be performed, and antimicrobials and intravenous immunoglobulin (IVIG) therapy should be administered to prevent long-term complications and mortality associated with this syndrome [2]. Therefore, we performed this cross-sectional cohort study in a large tertiary university hospital in China by analyzing the hospital and laboratory information system databases to systemically retrospectively identify GS patients in the First Affiliated Hospital of Wenzhou Medical University from Apr 2012 to Apr 2020. The clinical, laboratory, treatment, and outcome data for confirmed and suspected GS patients were collected and analyzed. To the best of our knowledge, this is the first and largest report of GS patients in Zhejiang, China. Each case had unique features in terms of the clinical presentation, laboratory results, and treatment duration.

## Methods

### Study design and data collection

This study was a retrospective investigation of 181 thymic or mediastinal tumor patients. All immunoglobulin test results of these patients ordered in the First Affiliated Hospital of Wenzhou Medical University from Apr 2012 to Apr 2020 were systemically reviewed. In 2019, the Wenzhou district of Zhejiang Province had a population of 8.2 million people. The concentrations of IgG (reference values from 7.0 to 16.0 g/L), IgM (reference values from 0.4 to 2.3 g/L), and IgA (reference values from 0.7 to 4.0 g/L) were measured by nephelometry (Siemens BN II) or Beckman Coulter IMMAGE800 [IgG (reference values from 7.51 to 15.6 g/L), IgM (reference values from 0.46 to 3.04 g/L), and IgA (reference values from 0.82 to 4.53 g/L)]. In brief, patients who repeatedly had IgG levels less than the reference value were regarded as potentially immunodeficient subjects. Additionally, the investigated lymphocyte subsets included B cells (reference values from 5 to 18%), CD3+ T cells (reference values from 60 to 79%), CD4+ T cells (reference values from 34 to 52%), and CD8+ T cells (reference values from 21 to 39%). All these lymphocyte subsets were detected by flow cytometry, and the data were extracted from the hospital information system. Patients with a diagnosis of any thymoma or anterior mediastinal tumor with hypogammaglobulinemia and low or absent B cells were included. The clinical manifestations of only thymoma patients with hypogammaglobulinemia were reviewed in detail. Survival data for all GS patients were documented by phone in July 2020. This study was approved by the First Affiliated Hospital of Wenzhou Medical University Ethics Committee.

## Results

### Forty percent of GS patients had not received a timely diagnosis in a tertiary university hospital

**Fig. 1:**
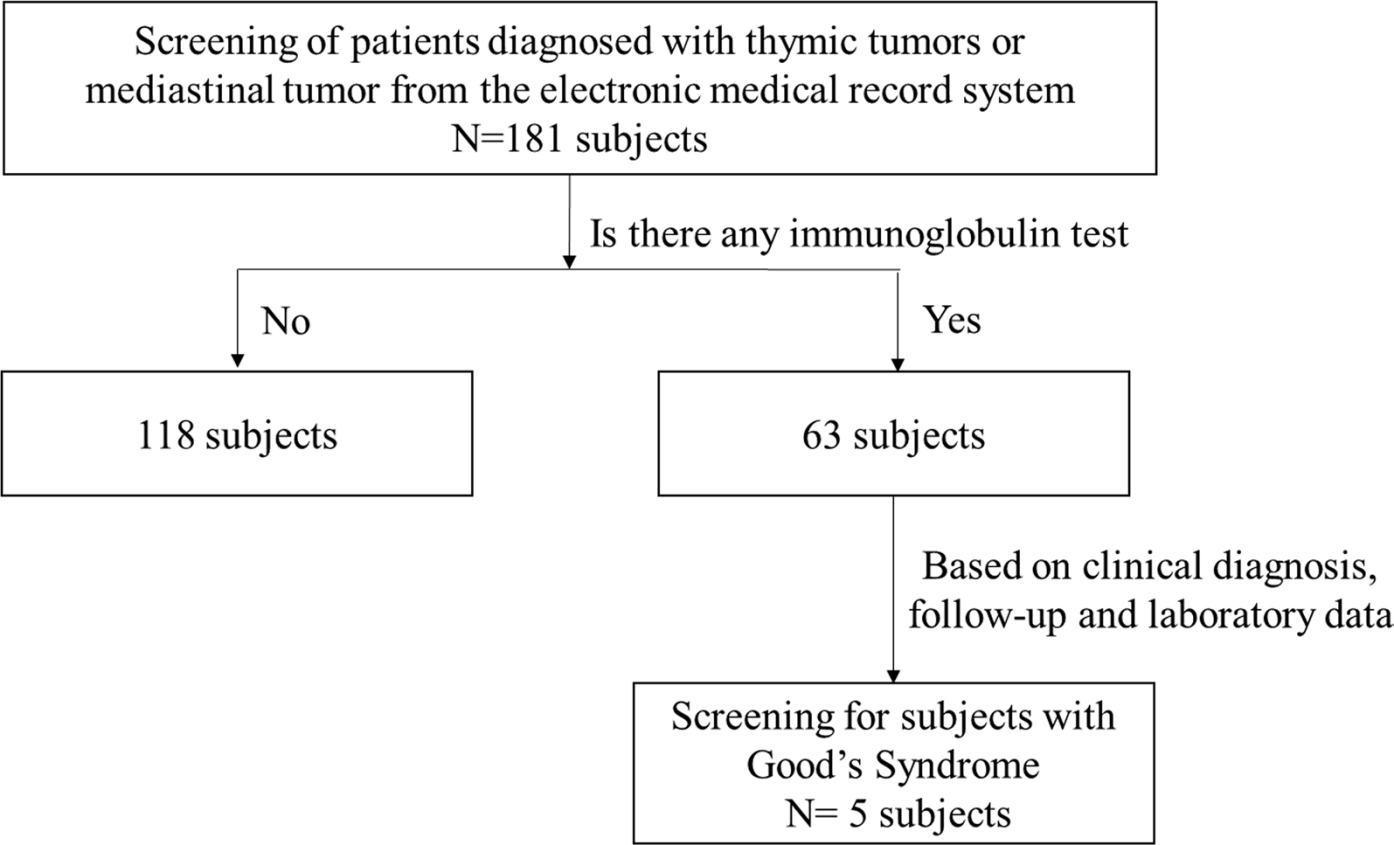
Flow chart of the selection of the 5 patients with Chinese Good's syndrome (thymoma and hypogammaglobulinemia) in a university hospital

GS is a rare secondary immunodeficiency disease. Due to its rarity, the diagnosis of GS is often missed. Thus, we used the electronic medical record system to retrospectively screen 181 thymoma or mediastinal tumor patients from Apr 2012 to Apr 2020 in a tertiary university hospital in Wenzhou, China. Among the 181 thymoma patients, 63 had undergone immunoglobulin tests; the other 118 thymoma patients had not undergone immunoglobulin tests (Fig. 1). Among these patients, 5 thymoma patients with hypogammaglobulinemia were identified; 3 patients had confirmed diagnoses of GS (Table 1), and the other 2 had not been diagnosed with GS (Table 1). A retrospective review of the clinical characteristics, laboratory results, and follow-up data for these 2 undiagnosed patients confirmed the diagnosis of GS. Interestingly, none of these 5 patients had autoimmune diseases. In the years after the diagnosis of hypogammaglobulinemia with mild symptoms, all patients received IVIG only intermittently. As the course of the disease progressed, the clinical symptoms of all patients worsened, but the symptoms of all patients partly resolved with IVIG. However, 4 patients died.

**Table 1:** Characteristics of the patients with Good's syndrome

Characteristics	Case 1	Case 2	Case 3	Case 4	Case 5
Age at diagnosis	43	54 (died in 2019)	55 (died in 2020)	70 (died in 2020)	57 (died in 2014)
Sex	M	M	M	F	M
Operation time	2016	2015	None	2016	2003
Thymoma histology	A	AB	B2	AB	AB
B cells (5–18%)	3.1↓	0.5↓	0.6↓	3.7↓	4.4↓
T cells (60–79%)	93.4↑	93.1↑	88.3↑	93.6↑	87.8↑
CD4 + T cell (34–52%)	17.5↓	13.7↓	8.4%↓	30.9↓	32.1↓
CD8 + T cell (21–39%)	65.4↑	63.5↑	74.7%↑	57.3↑	49.8↑
IgG (7.0–16.0 g/L or 7.51–15.6 g/L)	0.51↓	1.59↓	1.34↓	1.9↓	0.82↓
IgA (0.7–4.0 g/L or 0.82–4.53 g/L)	< 0.0667↓	< 0.26↓	0.25↓	< 0.0667↓	0.13↓
IgM (0.4–2.3 g/L or 0.46–3.04 g/L)	0.15↓	< 0.17↓	< 0.17↓	0.09↓	0.08↓
Clinical symptoms	Cough, expectoration, chest tightness, diarrhea	Cough, expectoration, fever, diarrhea	Skin and mucosal abscesses, cough, dyspnea	Cough, diarrhea, abdominal discomfort	Fever, cough
Main location of infection	Respiratory tract	Respiratory tract	Respiratory tract	Respiratory tract, intestinal tract	Respiratory tract
Severity of infection	Mild	Severe	Severe	Mild to Severe	Severe
Main comorbidities^a^	Bronchiectasis, pneumonia	Pneumonia, bronchiectasis, COPD, ARDS, respiratory failure	Pericardial placeholder, COPD, interstitial pneumonia	Oral lichen planus, intestinal infection, urinary tract infection	Interstitial pneumonia, pulmonary tuberculosis
Pathogen^b^	*C. difficile*	CMV, RuV, CVB, Aspergillus, SPn, ECl, Cal, Aba	CMV, Pc	CMV, Hp	Cal, TB
Treatment^c^	IVIG, Antibiotic	IVIG, ALB, Antibiotic, Antifungal, Methylprednisolone	IVIG, Antibiotic, Methylprednisolone	IVIG, ALB, Antibiotic	IVIG, Antibiotic, Methylprednisolone, Antiviral
Radiotherapy	No	No	Yes	No	Yes
Chemotherapy	No	No	Yes	No	No
Live status	Survived	Died	Died	Died	Died
Cause of death	None	Lung infection, coronary heart disease	Lung infection	Severe pneumonia, septic shock and respiratory failure	Septic shock, multiple organ failure

Note: B cells, CD4+ T cells, IgG, IgA, and IgM had the lowest test results. T cells and CD8+ T cells had the highest test results

^a^COPD: chronic obstructive pulmonary disease; ARDS: acute respiratory distress syndrome

^b^*C. difficile: Clostridium difficile*; CMV: cytomegalovirus; RuV: rubella virus; CVB: Coxsackie virus; SPn: *Streptococcus pneumonia*; ECl: *Enterobacter cloacae*; Cal: *Candida albicans*; Aba: *Acinetobacter baumannii*; Pc: *Pneumocystis carinii*; Hp: *Helicobacter pylori*; TB: *Tubercle bacillus*

^c^IVIG: Intravenous immunoglobulin; ALB: Intravenous albumin

### Two diagnosed GS patients presented with recurrent cough and expectoration

Case 1 was a 47-year-old man who initially presented with recurrent cough and expectoration for 4 months and chest tightness for 2 weeks in 2016 (Table 1). After admission, computed tomography (CT) results revealed bronchiectasis and thymoma. In 2016, he underwent thymectomy, which identified type A thymoma. Laboratory results revealed low IgG (0.51 g/L) and B lymphocytes (3.1%). Then, the patient was treated with tazobactam sodium/piperacillin sodium, mucosolvan, and ceftazidime during hospitalization. His condition improved during hospitalization. Additionally, he developed paranasal sinusitis, which was confirmed by magnetic resonance imaging (MRI). He was frequently admitted to the outpatient clinic to receive IVIG treatment. He had stopped IVIG treatment for 14 months prior to admission for economic reasons and had not experienced any significant symptoms.

Case 2 was a 54-year-old man who underwent thymectomy in 2015 for an AB-type thymoma. He initially presented with recurrent cough and expectoration in 2013. Subsequently, he developed chronic obstructive pulmonary disease (COPD), which was diagnosed in 2017, and he received IVIG treatment only once. In subsequent years, he developed episodes of recurrent cough and expectoration in addition to COPD, and these symptoms were partly resolved with antibiotics (sulperazone, meropenem, compound sulfamethoxazole, caspofungin, voriconazole) and methylprednisolone during hospitalization. Laboratory results revealed low IgG (1.59 g/L) and B lymphocytes (0.5%) in 2017 (Table 1). However, IVIG was only administered to this patient during hospitalization. The patient died of lung infection and coronary heart disease in 2019.

### One diagnosed GS patient presented with recurrent skin abscesses and dyspnea

Case 3 was a 57-year-old man who experienced dyspnea and a recurrent skin abscess for more than 10 years, and thymoma was confirmed by pathological cytology of the pleural fluid. Radiotherapy and chemotherapy with unknown drugs were administered in Italy in 2010 (Table 1). In 2018, this patient was admitted to our hospital because of complaints of recurrent cough and dyspnea, and chest CT revealed multiple occupied spaces in the mediastinum, with compression of the superior vena cava. Subsequent ultrasound bronchoscope-guided mass biopsy revealed that the secondary malignant tumor of the pericardium originated from a type B2 thymoma. After a series of examinations, *Pneumocystis carinii* infection, cytomegalovirus pneumonia, and interstitial lung disease were diagnosed, and antibacterial (tazocin, sulperazone, moxifloxacin, compound sulfamethoxazole), antifungal (caspofungin, voriconazole), and antiviral drugs (ganciclovir) and methylprednisolone were administered during hospitalization. Laboratory results revealed low IgG at 1.34 g/L and B lymphocytes at 0.6% in 2018. GS was diagnosed based on his symptoms and laboratory results. In subsequent years, he presented with recurrent skin ulcers, mouth ulcers, and keratitis, and these symptoms were partly resolved by IVIG. Unfortunately, this patient died of lung infection in 2020 during the preparation of this manuscript (Table 1).

### One undiagnosed GS patient presented with recurrent skin abscesses, oral lichen planus, pneumonia, and diarrhea

Case 4 was a 70-year-old woman who initially presented with recurrent oral lichen planus and skin abscesses on the right hand and left leg since 2007; the abscesses were treated with dexamethasone and vitamin B2 in a local hospital. In 2015, the patient was admitted to the hospital for acute lower respiratory infection. During this admission, routine chest CT revealed a mediastinal mass, and she underwent thymectomy in 2016 for an AB-type thymoma. After thymectomy, she experienced recurrent diarrhea, but the stools were negative for bacterial and parasitological pathogens. Gastroscopy revealed chronic, nonatrophic gastritis fundic polyps, and colonoscopy revealed ulcerative lesions in the colon. Subsequently, this patient had lost nearly 25 kg in weight. The basic laboratory test results showed the following in the patient’s peripheral blood: IgG, 1.90 g/L; IgM, 0.09 g/L; IgA, < 0.07 g/L; and B cells, 3.7%. Then, she was treated with panipenen/betamipron, levofloxacin, octreotide, amino acid, mucosolvan, and other antidiarrheal and intestinal flora regulation drugs during hospitalization. Interestingly, she repeatedly received albumin but not IVIG in a local hospital (Table 1). During follow-up, we educated this patient about GS and the advantages of IVIG for boosting immunity; the patient followed our suggestion and received IVIG in a local hospital. Unfortunately, this patient died of severe pneumonia, septic shock, and respiratory failure in 2020 during the preparation of this manuscript.

### One undiagnosed GS patient presented with recurrent pneumonia, tuberculosis, and granulomatous epididymitis

Case 5 was a 57-year-old man who initially presented with a tumor in the anterior mediastinum; he underwent thymectomy in 2003 for a type-AB thymoma. The right epididymis was surgically removed because a nodule in the right testis was found in 2005; pathology revealed that it was granulomatous epididymitis. Left lower pulmonary wedge resection was performed when a left pulmonary nodule was found in 2009, and pathology revealed left lower pulmonary tuberculosis. Then, standard antituberculosis therapy was initiated. Subsequently, the patient was repeatedly admitted to our hospital for recurrent pneumonia and interstitial pneumonia. The basic laboratory test results showed the following in the patient’s peripheral blood: white blood cells (WBCs), 0.75 × 10^9^/L; IgG, 0.82 g/L; and B cells, 4.4%. Then, he started to receive treatment with imipenem and cilastatin sodium, sulperazone, moxifloxacin, compound sulfamethoxazole, methylprednisolone, and ganciclovir during hospitalization. However, this patient was not diagnosed with GS (Table 1). Unfortunately, this patient died of septic shock and multiple organ failure in 2014.

## Discussion

To the best of our knowledge, clinical immunology services became available in Hong Kong in 2016, and at that time, there were no specialist immunology services for adult immunodeficiency in mainland China. General awareness regarding the care of immunodeficient adult patients is still inadequate in China [4]. GS and thymoma-associated immunodeficiency are rare clinical entities that are often presumed to be common variable immunodeficiency due to the lack of awareness and recognition of this syndrome. This syndrome often goes unrecognized if a thymoma is not detected. Defects in cell-mediated immunity are important causes of increased susceptibility to bacterial infections by encapsulated organisms and opportunistic viruses and fungi [5]. Further investigation of the immune system, including the detection of hypogammaglobulinemia and low or absent B cells, corroborates the diagnosis of GS. Current treatment for GS involves immunoglobulin replacement to maintain adequate trough IgG values, which can prevent long-term complications and reduce mortality.

In a large retrospective GS study in 2017, 47 GS patients from 27 studies were reported in China [3]. The initial clinical presentations varied. Sinopulmonary infection (74%) was the most common manifestation, followed by skin infection (10%) and intestinal tract infection (10%). Diarrhea was present in 36% of the patients, and autoimmune manifestations were present in 36% of the patients [3]. In this study, infections were identified in 100% of the hospitalized GS patients, suggesting that infection was the most frequent cause of hospitalization for GS patients [6].

In another large analysis of the clinical and laboratory features of 78 GS patients in the UK [2], 74 patients (95%) presented with infections, 35 patients (45%) had bronchiectasis, 7 patients (9%) had chronic sinusitis, and only 8 patients (10%) had serious invasive fungal or viral infections. Twenty (26%) patients suffered from autoimmune diseases (pure red cell aplasia, hypothyroidism, arthritis, myasthenia gravis, systemic lupus erythematosus, and Sjögren’s syndrome) [2]. Morbidity in GS patients is related to these infectious and autoimmune complications [2]. In contrast, none of the 5 GS patients in this study had autoimmune diseases. Diarrhea is a common symptom, and the etiology is straightforward in many cases. However, a common etiology was not identified in case 4; the patient had lost nearly 25 kg without IVIG treatment. Moreover, recurrent diarrhea, therapeutic resistance, secretory diarrhea, and even recurrent giardiasis have been reported in GS patients [7, 8]. Alternatively, the sole presentation of GS may mimic that of Crohn's disease [9]. Therefore, GS should be considered in the differential diagnosis if patients persistently present with chronic diarrhea [10].

Lichen planus has frequently been documented to be associated with GS [11–14]. In this study, case 4 presented with recurrent oral lichen planus for unknown reasons. The thymoma finding was incidental.

Moreover, vulvovaginal-gingival lichen planus is a distinct variant of lichen planus and may also be present in patients with GS [12]. Therefore, recurrent oral erosive lichen planus may indicate thymoma with secondary immunodeficiency [13]. It is important for dermatologists to recognize the clinical characteristics of patients with both lichen planus and thymoma with immunodeficiency [14].

Recurrent skin abscesses are frequently reported in patients with chronic granulomatous disease, leucocyte adhesion molecule deficiency, severe congenital neutropenia, and hyper-IgE syndrome [15]. In this study, recurrent skin abscesses were present in 2 GS patients. However, in the majority of adult thymoma patients, this clinical presentation does not indicate an underlying immune deficiency. Nevertheless, recurrent mucocutaneous abscesses can be associated with significant morbidity and long-term complications, including scarring and fistula formation, and they may be associated with an underlying immune-mediated disease. This study highlights that physicians treating patients with recurrent superficial abscesses should focus on the differential diagnosis, investigation and management of primary or secondary immunodeficiency.

The ideal treatment for GS is Ig replacement, and IVIG is recommended as a means of maintaining appropriate IgG levels in all GS patients [1]. It can significantly improve infection control, reduce hospitalization, and decrease the use of antibiotics if these GS patients have hypogammaglobulinemia [1, 3]. In the current study, all the patients received irregular IVIG, and immunodeficiency was partly resolved by IVIG. However, in the early stages of GS, all the patients received irregular IVIG due to economic reasons. As opportunistic infections increased in frequency, the patients began to receive IVIG regularly, consisting of 4–6 IVIG treatments in one hospitalization with a daily dose of 10 g each time, and their symptoms were significantly or partly resolved. Therefore, we highlight that although these GS patients did not initially present with infection, IVIG should be regularly administered to prevent complications by maintaining adequate IgG levels. Moreover, thymectomy is usually recommended in all patients with thymoma to prevent locally invasive growth and the metastasis of tumor cells. However, thymectomy is usually ineffective at improving immunodeficiency in GS patients, and it might worsen hypogammaglobulinemia in rare cases [16].

Moreover, in view of the rarity of GS and the lack of diagnostic guidelines, many GS patients may be missed. In addition to the most common manifestations, such as sinopulmonary infection, other skin or intestinal tract infections may not be recognized as being relevant. Moreover, in this study, recurrent skin abscesses, recurrent oral lichen planus, diarrhea, tuberculosis and granulomatous epididymitis were also found to be accompanying manifestations in GS patients. Therefore, examining concurrent Ig results could significantly improve the rate of diagnosis of GS. However, not all patients with thymomas and hypoimmunoglobulinemia can be diagnosed with GS, and other causes of hypoimmunoglobulinemia should be considered, such as autoimmune diseases, malignant tumors, and the usage of immunosuppressants, radiotherapy, or chemotherapy. Hypoimmunoglobulinemia may be transient when these causes are withdrawn. If these causes are not considered and withdrawn, patients may be misdiagnosed with GS and receive unnecessary IVIG treatment for the rest of their lives. In the present study, the patients were all negative for antinuclear antibodies, tumor markers, and immunosuppressant therapy. CT and B-ultrasound were performed to detect tumors, TpoAb and TgAb were tested to detect autoimmune thyroid disease. Other clinical manifestations were carefully reviewed, and the patients were found to be free from autoimmune diseases and other malignant tumors. Although case 3 had a history of chemotherapy and radiotherapy, and case 5 had a history of radiotherapy, the possibility of other causes was ruled out because hypoimmunoglobulinemia occurred prior to chemotherapy and radiotherapy and long-term follow-up. In addition, 2 patients had not been diagnosed with GS. The lack of awareness and recognition of this syndrome may have led to the 2 missed diagnoses upon follow up with the doctors in charge of these patients.

This study has certain limitations, as not all thymomas underwent immunologic evaluation. GS is most likely underreported since it may go unnoticed when thymoma is not considered clinically.

In conclusion, clinical acumen and an increased awareness of the clinical and immunological profiles of GS are needed to improve early diagnosis, which would promote improved therapeutic effects [3]. Moreover, GS should be considered as a differential diagnosis if patients present with chronic diarrhea, recurrent lichen planus, or recurrent infection. Additionally, we highlight that although these GS patients did not initially present with infection, IVIG should be regularly administered to maintain adequate IgG levels to prevent complications and reduce morality.

## Data Availability

The datasets used or analyzed during the current study are available from the corresponding author on reasonable request.

## Abbreviations

GS: Good's syndrome
IVIG: Intravenous immunoglobulin
IgG: Immunoglobulin G
IgM: Immunoglobulin M
IgA: Immunoglobulin A
CT: Computed tomography
MRI: Magnetic resonance imaging
COPD: Chronic obstructive pulmonary disease
WBCs: White blood cells
ARDS: Acute respiratory distress syndrome
*C. difficile*: *Clostridium difficile*
CMV: Cytomegalovirus
RuV: Rubella virus
CVB: Coxsackie virus
SPn: *Streptococcus pneumonia*
ECl: *Enterobacter cloacae*
Cal: *Candida albicans*
Aba: *Acinetobacter baumannii*
Pc: *Pneumocystis carinii*
Hp: *Helicobacter pylori*
TB: *Tubercle bacillus*
